# Development and validation of the questionnaire “Spiritual Needs in Palliative Care” in Finnish

**DOI:** 10.1017/S1478951526102168

**Published:** 2026-04-07

**Authors:** Raimo Goyarrola, Annamarja Lamminmäki, Suvi-Maria Saarelainen, Eeva Rahko, Kristiina Tyynelä-Korhonen, Kaisa Rajala, Mira Huhtala, Sofia Miinalainen, Reino Pöyhiä

**Affiliations:** 1 School of Medicine, University of Eastern Finland, Kuopio, Finland; 2 Department of Oncology, University Hospital, Kuopio, Finland; 3 School of Theology, Philosophical Faculty, University of Eastern Finland, Joensuu, Finland; 4 Department of Oncology, University Hospital, Oulu, Finland; 5 Palliative Care Center, Päijät-Häme Wellbeing Services County, Lahti, Finland; 6 Hospital Services of the Wellbeing County of Vantaa and Kerava, Vantaa, Finland; 7 Palliative Center and Department of Oncology, Turku University Hospital and University of Turku, Turku, Finland; 8 Palliative Center, Turku University Hospital and University of Turku, Turku, Finland; 9 School of Medicine, Department of clinical medicine, University of Eastern Finland, Kuopio, Finland

**Keywords:** Spiritual care, palliative care, spiritual needs, questionnaire, validation, Finland

## Abstract

**Objectives:**

Spiritual care is a core element of palliative care, addressing religious, spiritual and existential concerns and enhancing quality of life. In Finland, systematic assessment of patients’ spiritual needs is limited due to the lack of a validated instrument. This study aimed to develop and psychometrically validate the *Spiritual Needs in Palliative Care* (SNPC) questionnaire for Finnish palliative care patients.

**Methods:**

A prospective, multi-phase validation study, included item generation, expert review, pilot testing, and psychometric evaluation. Content and construct validity, as internal consistency and Cronbach’s alpha values were assessed using explorative factor analysis (EFA). For convergent and divergent, validity Pearson’s correlations were calculated for Edmonton Symptom Scale (ESAS), WHO Performance Status Scale, and the Spiritual Well-being Questionnaire (EORTC QLQ-SWB32).

**Results:**

The SNPC included 28 items covering existential, emotional, religious, and spiritual distress domains, with sections for importance and fulfillment of each need. A total of 116 patients (mean age 71 years; 61.2% female; 88.8% with cancer)), were recruited from 10 oncology and palliative care units across Finland. EFA supported an 8-factor structure – Communication and Preparation for Death; Meaning and Continuity; Emotional and Inner Peace; Artistic and Quiet Comfort; Religious Needs; Ritual Participation; Freedom from Guilt and Shame; Fear and Survival – explaining 71% of variance, with good reliability (Cronbach’s α = 0.63–0.93). The most important needs were safety in care, peace of mind, and participation in care decisions, while religious rituals and visits by clergy were less important. Significant gaps emerged between perceived importance and fulfilment of needs, regarding being heard, hope, peace, and the presence of loved ones. Fulfilled spiritual needs correlated well with SWB32 but not with ESAS.

**Significance of results:**

The SNPC is a valid and reliable instrument for assessing the spiritual needs of Finnish palliative care patients. It could support systematic identification of unmet spiritual needs of palliative care patients.

## Introduction

Spirituality remains ontologically exclusively undefined. Large individual variety in comprehension and experience of spirituality, the relational nature, and sensitivity to time and cultural differences has been claimed for that (Saarelainen et al. 2020; Nissen et al. 2021; Dorenbaum- Fastlicht et al. 2025), which makes an exclusive definition of spiritual care challenging, too. The WHO considers, however, spiritual care as an inseparable part of palliative care which supports patients in reconnecting with their beliefs, values, and practices that give them meaning and strength. In practice, spiritual care often involves interventions facilitating listening to patients’ fears, hopes, and concerns, while fostering opportunities to find meaning and purpose in illness (Büssing 2021; WHO 2025).

In this context, spirituality is understood broadly and is not limited to religious affiliation. While religion refers to institutionalized beliefs and practices, spirituality encompasses how individuals relate to questions of meaning, purpose, connection, and coherence in life, including but not restricted to religious frameworks (Selman et al. 2007; Büssing et al. 2013b; Wierstra et al. 2023). Accordingly, spiritual needs may be present regardless of whether individuals identify as religious, spiritual, both, or neither (Nolan et al. 2011).

Furthermore, spiritual care may include presence, awareness, respect, empathy, compassion, and commitment (Edwards et al. 2010; Walker and Waterworth 2017; Morland et al. 2022) expressed through communication and sustained support (Vivat 2008; Minton et al. 2018; Moudatsou et al. 2020).

Spiritual needs can be considered as multidimensional concerns through which individuals seek meaning, purpose, connection, coherence, and inner peace (Bradford 2023; Wei et al. 2025). They include existential questions about identity, suffering, hope, and values, as well as relationships; for some individuals, religious beliefs and practices are also part of these needs (Büssing et al. 2025). Spiritual needs may also be related to being recognized as a unique person, seeking forgiveness, or expressing love (Hermann 2006; Lunder et al. 2011; Bermejo Higuera et al. 2013). Unlike physical, psychological, or social needs, spiritual needs address the worldview through which people interpret life, illness, and relationships (Büssing et al. 2021).

Spiritual needs often emerge in everyday interactions between patients and healthcare professionals (Cobb et al. 2012; Best et al. 2015). There is also a considerable amount of overlapping between spiritual and social needs (Lormans et al. 2021). As an example, meaningfulness in daily life: the home care personnel, outdoor activities and green spaces greatly influenced on the meaningfulness of life in a recent study examining elderly Finnish frail patients cared at home (Hemberg et al. 2022).

Addressing patients´ spiritual needs may relieve distress, foster resilience, enhance the quality of life at the end of life (Balboni et al. 2007; Puchalski et al. 2009; Phelps et al. 2012; Sinclair et al. 2015), and promote holistic care (Baldacchino 2015). Even the families of the patients facing life-threatening illness can experience consolidation when their closest ones´ spiritual needs are met in the care (Gijsberts 2022).

By contrast, unfulfilled spiritual needs are associated with distress and depression, hopelessness, loneliness, guiltiness (Schultz et al. 2017; Ordons et al. 2018), reduced physical well-being (Hui et al. 2011; Caldeira et al. 2013) and diminished quality of life (Winkelman et al. 2011) and satisfaction with care (Riklikienė et al. 2020).

Although clinical studies and systematic reviews have suggested many successful interventions for improving spiritual care (Hvidt et al. 2017; Pérez-Eizaguirre and Vergara-Moragues 2021; Warth et al. 2021; Huda et al. 2023; Valero-Cantero et al. 2024; Brungardt et al. 2024; Nassehi et al. 2024), unfortunately spiritual needs are rarely addressed in clinical practice (Gijsberts et al. 2019; Varner-Perez et al. 2024). Barriers for health-care professionals to recognizing spiritual needs include different concepts of spirituality, absence of spiritual care guidelines; shortage of time; and insufficient training and education on spiritual issues, shame of approaching spirituality and lacking spiritual or religious literacy (Rushton 2014; Hvidt et al. 2017; Dellenborg and Enstedt 2023; Laranjeira et al. 2023; Costeira et al. 2024).

In the Nordic countries spiritual issues have been traditionally considered very individual matters, which seldom are discussed in care settings and remain poorly anchored in public health care (Viftrup et al. 2021). As an example, retrospective studies from Norway and Sweden have observed very few mentions about spiritual needs and care in nursing reports in palliative care (Gunhardsson et al. 2008; Hynnekleiv et al. 2024). Yet a recent large population-based questionnaire survey including 104,137 Danish adults observed that over 80% of responders had experienced spiritual needs (Stripp et al. 2023).

Contemporary qualitative research has provided insight for detecting patients´ spirituality and spiritual issues nonverbally in behavioral patterns and care facilities (Toivonen et al. 2018; Penman 2021; Voetmann et al. 2022). Health-care professionals´ communication with patients about the spirituality can importantly be facilitated with specific questionnaires such as the Spiritual Needs Questionnaire (SpNQ) (Büssing et al. 2010) and the Spiritual Needs Assessment for Patients (SNAP; Sharma 2012) developed for assessment of needs in palliative care. Their use requires naturally that patients are able to communicate verbally (Büssing et al. 2013b; Moeini et al. 2018; Zhao et al. 2019; Brandstötter et al. 2025; Hagelin et al. 2025). Also, these questionnaires must be culturally adapted.

Although SpNQ was recently validated in Danish (Stripp et al. 2022), a nationally designed instrument for palliative care has not been developed in the Nordic context (Benzein and Berg 2004; Asgeirsdottir et al. 2017; Stripp et al. 2023). Spirituality has been overall studied very little in Finnish palliative care, but the previous Finnish qualitative studies have detected insufficient spiritual care in palliative units. Spiritual issues are not met enough well and that spiritual care skills of the nurses are not sufficient in palliative care (Lammi et al. 2001; Selman et al. 2018; Toivonen et al. 2018). Yet the importance of spirituality and the relationship of higher spiritual well-being and general quality of life and psychological well-being has been documented also among Finnish patients (Norberg et al. 2015; Saarelainen et al. 2020; Goyarrola et al. 2024). Clearly, there is a need for a culturally grounded tool for palliative care professionals to better identify and address patients’ spiritual needs, thereby strengthening the provision of truly holistic care.

## Methods

### Aim

The study aimed to develop a novel instrument for assessing the spiritual needs of palliative care patients. The finalized questionnaire was created in Finnish, with an official English translation to support broader applicability and cross-cultural use.

### Study design for elaboration and validation

**Figure 1.**
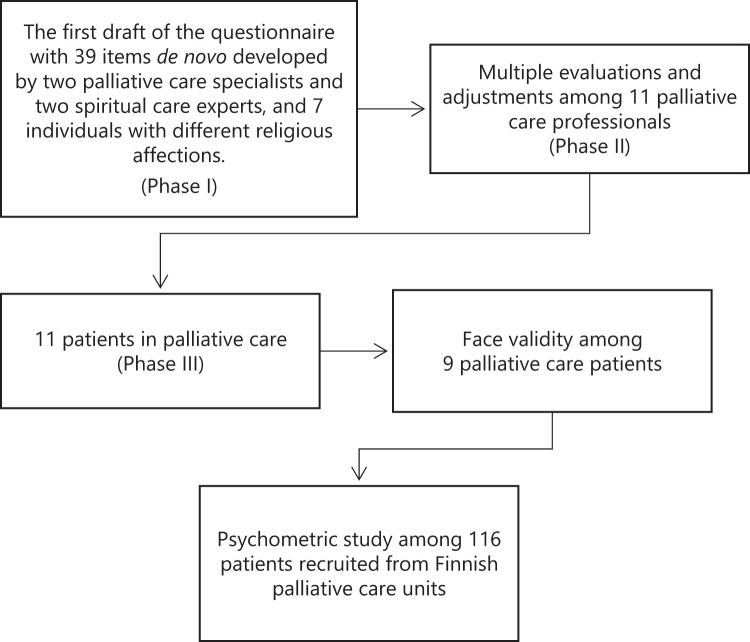
Flow chart of the developmental process of the Finnish questionnaire of spiritual needs. → = “leading to”.

The questionnaire was developed, pre-tested, and validated using a prospective study design. Development began in 2020 and proceeded in three phases (Fig. 1). Throughout the process, the core research expert team (RG, AL, SS, RP) the expert team held monthly online meetings to refine the questionnaire. Thus, the role of the expert research team was vital during the entire development process.

### Questionnaire development

#### Phase 1

An initial pool of 39 items representing spiritual needs was developed by the research main group of two palliative care physicians and two spiritual care experts, on the basis of previous international studies (Bussing 2010; Sharma 2012), a recent Finnish qualitative study (Saarelainen et al. 2020) and their long-time experience in discussions with palliative care patients. Items were grouped into psychological (*n* = 14), existential/spiritual (*n* = 17), and religious (n = 8) domains. After refinement with input from 3 additional medical professionals, the questionnaire was revised to 36 items, evenly divided into psychological, existential, and religious needs (12 items in each). The structure, designed to assess both met and unfulfilled needs, was reviewed by representatives of 6 religious traditions in Finland and 1 non-affiliated individual (*n* = 7). Using a 0–5 rating scale, participants evaluated each need’s importance and could mark indisputable items. A cut-off score of 21 (≥60% of maximum 35 points) determined item acceptance. Participants were also asked to select their prioritized items.

#### Phase 2

The revised questionnaire was evaluated by 11 health-care professionals experienced in palliative or long-term care, who assessed its content and structure using the same Likert-type scale as in the first phase. Content validity was measured with the Content Validity Index (CVI), calculated as the proportion of essential ratings (Yusoff 2019). Items with a CVI of 0.6–0.8 were reconsidered after discussion, while those below 0.6 were excluded. Participants could also suggest new items and provide qualitative feedback on clarity, wording, and response format. Based on this review, the Finnish version was revised and expanded to 27 items.

#### Phase 3

The questionnaire was pilot tested with 11 patients through individual pre- and post-completion interviews. Interviews explored clarity, comprehensibility, and sensitivity, asking whether any text was difficult, confusing, offensive, or distressing. Participants were also invited to suggest modifications to improve clarity and acceptability. Four team members re-evaluated the final questionnaire.

### Validation of the final questionnaire

The final *Spiritual Needs in Palliative Care* (SNPC) questionnaire comprises 28 items on a 5-point verbal rating scale that assess both the importance (“*How important is this need to you*?”: 0 = I cannot say. 1 = Not at all important. 2 = Somewhat important. 3 = Fairly important. 4 = Very important), and fulfilment (“*How is this need being met for you at the moment?”:* 0 = I cannot say. 1 = Not at all. 2 = To some extent. 3 = Fairly well. 4 = Very well). Items were reorganized using an online pseudorandom number generator. For each need, participants rated its importance and the extent to which it was satisfied and were additionally asked to indicate any other needs.

### Face validity

Face validity of the final SNPC questionnaire was assessed with 9 palliative care patients. Each of them received an explanation of the questionnaire beforehand, and was interviewed afterward to evaluate its comprehensibility, relevance, and acceptability.

### Testing and the population

The final questionnaire was administered to 135 patients from palliative care units across Northern, Eastern, Western, and Southern Finland. Eligibility required adequate cognitive and linguistic ability in Finnish and diagnosis of serious, incurable life-limiting and progressive disease and receiving or being eligible for receiving palliative care. The eligibility refers to such patients who didn´t yet have a formal palliative care decision but were consulted for a palliative care specialist because of approaching palliative care at the time of filling of the questionnaire. Alongside spiritual needs, the instrument assessed demographics (age, gender), clinical status (diagnosis, palliative care status, past-week health), quality of life, functional capacity, and religious or spiritual affiliation.

### Validity and internal consistency

Construct validity and internal consistency were studied using exploratory factor analysis (EFA; Mvududu and Sink 2013).

For Convergent/divergent validity associations between SNPC scores, spiritual well-being, quality of life, and symptom burden were examined using the Spiritual Well-Being Questionnaire (EORTC-SWB32) (Vivat et al. 2017), the Edmonton Symptom Assessment System (ESAS; Hui and Bruera 2017), and the WHO Performance Status Scale (Zubrod et al. 1960). The SWB32 is a 32-item, cross-culturally validated tool measuring five dimensions of spiritual well-being (self, others, something greater, existential concerns, God); its Finnish version has been recently validated (Goyarrola et al. 2023). The ESAS rates 13 common symptoms in advanced illness on a 0–10 scale, while quality of life and perceived health are assessed on a 7-point scale (1 = very poor, 7 = excellent). The WHO Performance Status Scale evaluates daily functioning from 0 (fully active) to 4 (bedbound) and is widely used in oncology and palliative care.

### Data collection and statistical analysis

Data collection, anonymization, and digitalization took place in 2024–2025. Descriptive results are reported as means with standard deviations (SD) or percentages. Statistical analyses included Pearson’s correlation coefficients, independent samples *t*-tests. Correlation strength was defined as >0.5 strong, 0.3–0.5 moderate, 0.2–0.29 weak, and <0.2 negligible.

EFA was performed using principal axis factoring with varimax rotation to assess construct validity (Fabrigar et al. 1999; Costello and Osborne 2005). Suitability for factor analysis was confirmed with the Kaiser–Meyer–Olkin (KMO) measure and Bartlett’s test of sphericity (Kaiser 1974). Internal consistency was evaluated using Cronbach’s alpha.

Statistical significance was set at *p* < 0.05 (*) and *p* < 0.01 (**). All analyses, including factor analyses, were performed using IBM SPSS Statistics version 27.0.1 (Armonk, NY: IBM Corp.).

## Results

### Questionnaire development

#### Phase 1

In the preliminary 39-item questionnaire, 1 item (“to be complete and safe”) was removed, and 4 were consolidated into 2, yielding 36 items. The mean ranking (SD) was 27 (3.8).

The highest-ranked needs (31–35) emphasized both existential and relational dimensions, such as *peace of mind, participation in care decisions in line with personal convictions, and sharing thoughts and experiences with loved ones.* Needs related to explicitly religious practices, such as *funeral rites or finding one’s relationship with God or something higher,* were also among the most valued.

The lowest-ranked needs (10–21) included *To dive into the beauty of nature, To keep in touch with other patients suffering from similar illnesses, To find meaning in the experience of illness, To know how to relax and manage stress, To overcome feelings of rejection, vulnerability, or isolation, and To create reconciliation of old disputes, hurts, or resentments with family or friends.*

Some elements of these items were merged into others, and further consolidation reduced the questionnaire to 25 items, with revised wording. Figure 1 summarizes the development process.

#### Phase 2

The lowest-rated items were *Finding meaning in my illness, Participating in care decisions according to my beliefs, Experiencing the beauty of nature, Planning my own funeral,* and *Being free from shame or guilt* (ratings 2.93–4). Content CVI values ranged from 0.2 to 1, leading to the exclusion of *Finding meaning in the disease* (CVI = 0.2). In the second phase, the Finnish wording of all items was refined. Based on this feedback, two physicians and two humanities experts finalized a 27-item instrument. The questionnaire’s structure was rated entirely appropriate (CVI = 1); it includes two columns – one for the importance of each SN and another for its fulfilment – plus an open column for respondents to add other needs and descriptions.

#### Phase 3

One participant noted difficulty understanding items on *freedom from shame, guilt, and meaning in life*. Most needs were rated rather or very important (scores ≥3–4) in 88% of responses. *Discussing death, freedom from shame, and freedom from guilt* were rated moderately important (score = 2) in 10% of responses, while *prayer and meeting a faith leader* were considered unimportant by 2%. No new needs were suggested, and no items were reported as confusing, offensive, or distressing; no modifications were proposed. Based on expert team´s consensus, a new item – *Finding comfort in the arts (e.g., films, books, music)* – was added, yielding the final 28-item SNPC questionnaire.

### Validity and reliability of the final questionnaire

The nine patients confirmed the face validity of the questionnaire, reporting that it was comprehensible, relevant, and acceptable, with no difficulties encountered.

### Participants

Table 1 summarizes Participants’ demographic and clinical characteristics. Most of the patients had cancer diagnosis (88.8%), and 70.6% had a palliative treatment plan. The quality of life and spiritual well-being were rated as moderate to good, and more than half reported moderate functional capacity.

**Table 1.** The characteristics of the participants in the final validation part

Variable	Total
*Total participants*	116
*Gender*	
Female	71 (61.2%)
Male	45 (38.8%)
*Age (M/SD)*	
Female	70.77 (11.28)
Male	71 (10.64)
*Primary illness*	
Cancer	103 (88.8%)
Cardiac	6 (5.1%)
Respiratory	1 (0.8%)
Nervous	5 (4.3%)
Musculoskeletal	1 (0.8%)
*Secondary illness*	
Non-mentioned	75 (64.6%)
Cardiac	13 (11.2%)
Respiratory	11 (9.5%)
Nervous	2 (1.7%)
Endocrine	12 (10.3%)
Musculoskeletal	3 (2.6%)
*Palliative treatment goal*	
Yes	82 (70.6%)
No	21 (18.1%)
Unclear	13 (11.2%)
*General health status (very bad: 1/very good: 7)*	
1	2 (1.7%)
2	5 (4.3%)
3	12 (10.3%)
4	33 (28.4%)
5	43 (37%)
6	16 (13.8%)
7	5 (4.3%)
*General spiritual status (very bad: 1/very good: 7)*	
1	1 (0.8%)
2	5 (4.3%)
3	12 (10.3%)
4	30 (25.8%)
5	44 (37.9%)
6	18 (15.5%)
7	6 (5.1%)
*Capacity of activity (capable: 0/uncapable: 4)*	
0	14 (12%)
1	65 (56%)
2	15 (13%)
3	15 (13%)
4	7 (6%)
*Spiritual/religious*	
Lutheran	80 (68.9%)
Other Christian nominations	15 (12.7 %)
Buddhist	1 (0.8%)
No religious/but spiritual	8 (6.9%)
No religious/no spiritual	12 (10.3%)
*Living area in Finland*	
Northern part	18 (15.5 %)
Eastern part	25 (21,5 %)
Western part	20 (17.2 %)
Southern part	53 (45.7 %)

*M* = mean, SD = standard deviation.

### Content validity

**Figure 2.**
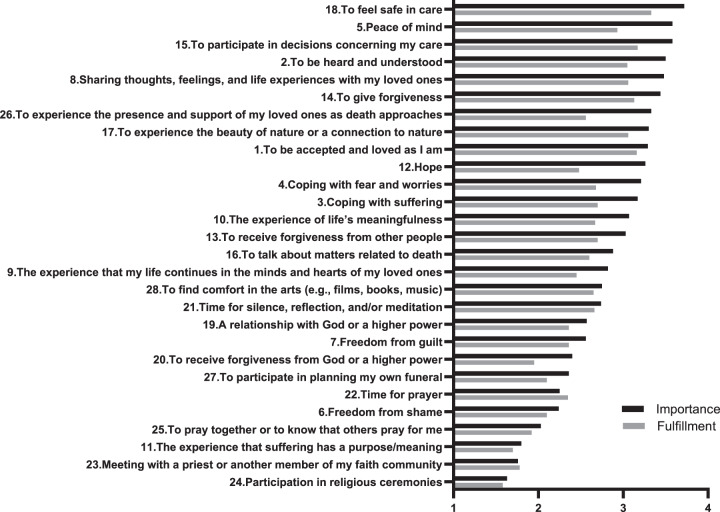
Means of Likert scales of importance (“*How important is this need to you*?”: 0 = I cannot say. 1 = Not at all important. 2 = Somewhat important. 3 = Fairly important. 4 = Very important), and fulfilment (“*How is this need being met for you at the moment?”:* 0 = I cannot say. 1 = Not at all. 2 = To some extent. 3 = Fairly well. 4 = Very well).

A total of 116 patients completed the finalized SNPC questionnaire; nine questionnaires with substantial missing data were excluded. Figure 2 shows the perceived importance and fulfilment of SNs, ranked from highest to lowest importance. The most highly rated needs for importance were *To feel safe in care (18), Peace of mind (5),* and *To participate in care decisions (15)*. The lowest-rated needs were *Participation in religious ceremonies (24), Meeting with a priest or another member of my faith community (23),* and *The experience that suffering has a purpose/meaning (11)*. For fulfilment, the highest-rated needs were *To feel safe in care (18)* and *To be accepted and loved as I am (1)*, while the lowest were *Participation in religious ceremonies (24)* and *The experience that suffering has a purpose/meaning (11)*. Significant discrepancies between importance and fulfilment were observed for *To be heard and understood (2), Peace of mind (5), Hope (12),* and *To experience the presence and support of my loved ones as death approaches (26).*

### Construct validity and internal consistency

Quantitative analysis (Table 2) provided descriptive statistics and response distributions for all items. Item-level analysis assessed ceiling and floor effects, defined as >95% of responses in two categories or <5% in any category. The number of factors was guided by statistical calculations and confirmatory graphs, with parallel analysis confirming the optimal latent constructs. They were named according to our primary theoretical conceptualization. Internal consistency was high across eight categories, with Cronbach’s alpha coefficients ranging from 0.627 to 0.927. The overall Cronbach’s alpha for the SNPC scale was 0.714.

**Table 2.** Exploratory Factor Analysis SNPC “Importance of the Needs” (Range 1–4). Com: Communalities; Rot: Varimax rotated factor matrix. *Extraction Method*: Principal Axis Factoring. *Rotation Method*: Varimax with Kaiser Normalization Kaiser–Meyer–Olkin Measure of Sampling Adequacy: 0.779. Bartlett’s Test of Sphericity *p*-value: 0.000. Bartlett’s Test of Sphericity Approx. Chi-Square: 1703.082; df 378; Sig. < 0.001. Those 8 factors: % of Variance Cumulative 70.997%

Spiritual needs dimension	Factor	Item	Mean	Com	Rot
**Existential (8 items)**	***4: Communication and preparation for death***	2. To be heard and understood	3.50	0.574	0.611
		16. To talk about matters related to death	2.88	0.484	0.628
	*Variance: 1.387 %*				
	*of Variance: 4.954*				
	*Cronbach´s α: 0.703*	1. To be accepted and loved as I am	3.29	0.564	0.512
		26. To experience the presence and support of my closest ones as death approaches	3.33	0.230	0.366
		27. To participate in planning my own funeral	2.36	0.425	0.406
	***5: Meaning and continuity***	9. The experience that my life continues in the minds and hearts of my closest ones	2.82	0.642	0.634
	*Variance: 1.288* *% of Variance: 4.602*	10. The experience of life’s meaningfulness	3.07	0.713	0.567
	*Cronbach´s α: 0.751*				
		8. Sharing thoughts, feelings, and life experiences with my closest ones	3.48	0.603	0.480
**Emotional and aesthetic (8 items)**	***3: Emotional and inner peace***	14. To give forgiveness	3.44	0.519	0.583
		13. To receive forgiveness from other people	3.03	0.536	0.559
	*Variance: 1.904*				
	*% of Variance: 6.801*				
		17. To experience the beauty of nature or a connection to nature	3.30	0.472	0.614
	*Cronbach´s α: 0.759*				
		15. To participate in decisions concerning my care	3.58	0.370	0.495
		18. To feel safe in care	3.72	0.531	0.513
		5. Peace of mind	3.58	0.533	0.487
	***7: Artistic and Quiet Comfort***	21. Time for silence, reflection, and/or meditation	2.74	0.575	0.519
	*Variance: 1.118*	28. To find comfort in the arts (e.g., films, books, music)	2.75	0.419	0.535
	*% of Variance: 3.994*				
	*Cronbach´s α: 0.642*				
**Religious (6 items)**	***1: Religious needs***	22. Time for prayer	2.25	0.771	0.814
	*Variance: 8.446*	25. To pray together or to know that others pray for me	2.03	0.712	0.811
	*% of Variance: 30.163*	20. To receive forgiveness from God or a higher power	2.40	0.923	0.791
	*Cronbach´s α: 0.927*	19. A relationship with God or a higher power	2.57	0.845	0.781
	***8: Ritual Participation***	24. Participation in religious ceremonies	1.63	0.899	0.326
	*Variance: 1.082*	23. Meeting with a priest or another member of my faith community	1.76	0.687	0.289
	*% of Variance: 3.864*				
	*Cronbach´s α: 0.900*				
**Spiritual distres (6 items)**	***2: Freedom from Guilt and Shame***	6. Freedom from shame	2.24	0.905	0.878
	*Variance: 3.426*	7. Freedom from guilt	2.56	0.687	0.739
	*% of Variance: 12.235*				
		11. The experience that suffering has a purpose/meaning	1.80	0.477	0.531
	*Cronbach´s α: 0.791*				
		12. Hope	3.26	0.546	470
	***6: Fear and survival***	4. Coping with fear and worries	3.21	0.705	0.796
	*Variance: 1.227*	3. Coping with suffering	3.17	0.380	0.413
	*% of Variance: 4.384*				
	*Cronbach´s α: 0.627*				

### Convergent/divergent validity of SNPC

Association of needs, SWB32, and ESAS are shown in Table 3. Existential, emotional, and religious needs were each significantly associated with their corresponding aspects of spiritual well-being in SWB32. In contrast, spiritual distress was linked to lower overall well-being. These associations remained independent of physical symptoms, including pain in ESAS, which showed no significant correlations with spiritual or emotional factors. We also found several significant associations between quality of life, WHO performance status, and SWB32 (Table 4). In contrast, correlations between the spiritual needs’ factors and overall symptom burden, as measured by ESAS were consistently low and non-significant across all dimensions.

**Table 3.** *Correlations SNPC factors/SWB32/ESAS (SN.Exi: Existential dimension of spiritual needs; SN.Emo: Emotional and esthetic dimension of SN; SN.Rel: Religious dimension of SN; SWB32. Exi: Existential items; SWB32. Emo: Emotional items; SWB32.Rel: Religious items; SWB32: item 32: Spiritual well-being; ESAS.WB*: Over-all well-being)

Groups of factors	SN.Exi	SN.Emo	SN.Rel	SN.dis
SN.Exi	1	0.596**	0.366**	0.607**
SN.Emo	0.596**	1	0.444**	0.534**
SN.Rel	0.366**	0.444**	1	0.282**
SN.dis	0.607**	0.534**	0.282**	1
SWB32.Exi	0.340**	0.351**	0.091	0.320**
SWB32.Emo	0.470**	0.449**	0.462**	0.310**
SWB32.Rel	0.324**	0.453**	0.630**	0.304**
SWB32	0.210*	0.166	0.213*	0.195*
ESAS.WB	0.074	0.092	0.138	0.057

** Correlation is significant at the 0.01 level (2-tailed).

* Correlation is significant at the 0.05 level (2-tailed).

**Table 4.** Correlations between spiritual well-being (SWB32), general condition (ESAS), functional status (WHO), and patient´s appreciation on own health state and quality of life. SWB32: Spiritual Well-Being Questionnaire; ESAS: Edmonton Symptom Assessment System; WHO: Performance Status Scale

Groups	SWB32	ESAS general condition	General state of health	Quality of life	WHO
SWB32	1	−0.124	0.158	0.243**	−0.090
ESAS_General Condition	−0.124	1	−0.428**	−0.465**	−0.306**
General health condition	0.158	−0.428**	1	0.820**	0.507**
Quality of life	0.243**	−0.465**	0.820**	1	0.437**
WHO	−0.090	−0.306**	0.507**	0.437**	1

** Correlation is significant at the 0.01 level (2-tailed).

## Discussion

This study describes the development and validation of the SNPC questionnaire, the first culturally adapted tool for assessing spiritual needs in Finnish palliative care patients. The SNPC demonstrated satisfactory content and construct validity, good internal consistency, and convergent validity with the EORTC-SWB32, as well as divergent validity with the ESAS. Strong correlations with the SWB32 indicate that the SNPC effectively captures key aspects of spiritual well-being. At the same time, the lack of association with symptom burden (ESAS) highlights that spiritual needs are distinct from physical and psychological symptoms.

### Main findings

Exploratory factor analysis identified eight domains encompassing existential, emotional-aesthetic, religious, and distress-related needs, reflecting the multifaceted nature of spirituality in palliative care (Selman et al. 2018), including meaning, connection, inner peace, and reconciliation (Puchalski et al. 2009; Vilalta et al. 2014). Notably, “Emotional and Inner Peace” and “Freedom from Guilt and Shame” emerged as distinct factors, capturing key psychological and existential dimensions (McClain et al. 2003; Cheng et al. 2023). Consistent with prior research, patients prioritized existential and emotional needs – such as hope, being heard, and the presence of loved ones – over formal religious practices (Balboni 2013). Needs related to rituals, clergy visits, or finding meaning in suffering were rated lowest, highlighting that some patients experience spirituality outside formal religious frameworks (Steinhauser et al. 2000; Büssing et al. 2013a).

Strong correlations but low mean scores in items reflecting traditional spirituality and religion (e.g., praying together, meeting a priest) suggested that while fulfilment is linked to the expression of these needs, they are not widely prioritized by all patients. Although less prominent overall, religious needs remained important for a substantial subgroup, highlighting the diversity of spiritual needs. These findings indicate a cultural shift in spiritual care toward relational, emotional, and meaning-centered domains in secularized societies such as Finland, consistent with international research (Büssing et al. 2010; Nygaard et al. 2022).

Emotional distress is common among patients with advanced cancer, and prior studies often show moderate correlations between symptom burden and psychological distress (Ferrell et al. 2017; Ascencio-Huertas et al. 2021). In our study, however, ESAS scores did not correlate significantly with distress measures. Distress may arise from psychosocial or spiritual factors beyond physical symptoms, and symptom control can reveal underlying needs that require attention to improve quality of life (Ostwal et al. 2021). Evidence suggests that spiritual care may support coping with pain rather than reducing its intensity (Hindmarch et al. 2022). Our findings highlight the distinct yet potentially overlapping nature of pain, spiritual distress, and emotional well-being. More comprehensive psychometric assessments and advanced analyses may reveal subtle or context-specific relationships that simple correlations cannot detect (Gonçalves et al. 2024).

In this study, we observed a gap between the high importance patients place on existential and emotional needs and their lower reported fulfilment. This underscores a persistent challenge in palliative care: although spiritual needs are widely recognized as essential, they are often inadequately addressed in practice (Koper et al. 2019; Jones et al. 2021). Provision of spiritual care in medical settings is consistently limited, even though patients – particularly those at the end of life – express a strong desire for holistic recognition of these needs (Ripamonti et al. 2018).

In our study, unmet needs related to reconciliation, forgiveness, guilt, and existential suffering were particularly concerning, as they can exacerbate anxiety, depression, and hopelessness (Büssing 2021). Addressing these sources of spiritual distress is essential, and health-care professionals should be able to recognize both religious and non-religious forms of spirituality and respond flexibly to individual concerns (Batzler et al. 2024). Some clinicians also reported discomfort or lack of confidence in addressing existential or religious issues, sometimes leaving patients to manage these concerns alone (Prieto-Crespo et al. 2024). This is not an exception (Taylor 2021). Barriers included staff shortages, inadequate training (O’Brien et al. 2019), and the perception that spirituality is too personal or falls beyond of the scope of professional responsibility (Vivat et al. 2023). There is a clear need for professional skills and training to identify and address patients’ spiritual needs effectively (Batstone et al. 2020; Büssing et al. 2025).

Significant gender differences were observed in existential and religious factors, with females scoring higher than males (existential: *M* = 61.08 vs. 46.80; religious: *M* = 62.55 vs. 46.79), indicating greater concern in these domains among women. No significant differences were found for emotional factors (females: *M* = 58.28; males: *M* = 49.77) or spiritual distress (females: *M* = 52.72; males: *M* = 57.20). These findings are consistent with previous reports that women more frequently express spiritual and existential concerns (Hermann 2007) and highlight the importance of individualized approaches considering demographic and cultural factors in spiritual care (Steinhauser et al. 2017).

### Strengths and limitations

This study has several strengths, including rigorous psychometric development, multicenter recruitment, and the involvement of both patients and professionals in validation and thorough psychometric analysis. The SNPC demonstrated strong internal consistency across most subscales and robust construct validity.

To our knowledge, it is the first validated tool for assessing spiritual needs in Finnish palliative care. These findings support the SNPC’s utility as a clinical and research instrument, addressing a previously noted barrier to culturally appropriate spiritual care in Finland and beyond. A major strength of the SNPC is its ability to adapt to cultural contexts. Unlike existing instruments such as the Spiritual Needs Questionnaire (SpNQ) (Büssing 2021), the SNAP (Sharma et al. 2012), and the Spiritual Concerns Checklist (SCC) (Michael et al. 2023), the SNPC was explicitly developed for the Finnish palliative context. Evidence from other cultural adaptations – e.g., SNAP in Chinese populations (Astrow et al. 2012) and SpNQ in Chinese (Büssing et al. 2013b) and Brazilian populations (Valente et al. 2018) – demonstrates that conceptualizations of spirituality vary widely across cultures, highlighting the importance of context-specific tools (Chen et al. 2018). However, it would be interesting to find out, how our new questionnaire would fit in another culture.

Our questionnaire has rather similar number of items as the most of the previous English ones (Galek et al. 2005; Büssing et al. 2010, Vilalta et al. 2014). A unique difference is that we have focused both on the importance and fulfilment of the needs.

We believe that the new Finnish questionnaire could serve as a screening tool, as a discussion aid for those palliative care professionals who do not feel comfortable with spiritual issues, as a research instrument and as a bench-marking tool in palliative care.

A strength of the current study is that this new tool complements well with our Finnish translation of the EORTC-SWB32 (Goyarrola et al. 2023), which lacks detailed inquiry of spiritual needs but rather assesses the spiritual wellbeing of a patient.

Several limitations should be noted. Our questionnaire was tested mostly among primarily older cancer patients with Finnish Lutheran background, which may limit its use for patients with other diagnoses, cultures, or spiritual affiliations. Furthermore, as researchers with variable but mainly Christian backgrounds, we acknowledge that our professional experiences and personal beliefs may influence how we interpret data. The team engaged in regular discussions and reflexions to critically examine the interpretations. Divergent viewpoints within the team were discussed and integrated to minimize individual bias. Some subscales, such as *Artistic and Quiet Comfort*, showed lower internal consistency, indicating a need for refinement. Further qualitative studies on lived religion show, religiosity and spirituality are inherently situational, embodied, relational, and contextual; therefore, the operationalization of the scale may have captured only a partial representation of the lived realities of SNPC(McGuire 2008; Ammerman 2014).

We did not formally assess the education levels of the responders, but in the preliminary assessments all participants had a professional education and the patients in the validation part represented well general well-educated Finnish population. No signs of difficulties in understanding the wordings were observed. The cross-sectional design precludes assessment of how spiritual needs change over time. Future longitudinal studies should evaluate the SNPC’s sensitivity to change and its predictive value for patient outcomes. We acknowledge as a limitation, that patients with cognitive decline, severe deterioration or fragility may not be able to respond to the whole questionnaire or even any part of it. Even in the care such patients, the questionnaire putatively might help palliative care staff or used only partly.

### Implications for practice and research

Clinically, these findings highlight the ethical and practical imperative to integrate spiritual assessment into routine palliative care. Addressing spiritual needs can enhance patient satisfaction and has been associated with improved emotional functioning, coping, and quality of life at the end of life. We believe that the new questionnaire could be used both in the clinics and studies of palliative care. We think that this questionnaire would be particularly useful to help the palliative care staff to acknowledge spiritual issues in clinical practice those palliative care professionals, who express difficulties regarding existential, spiritual and religious issues. The SPNC could be used both as a written document by the patient or a nurse or a physician as an aid for the professionals during the discussions with the patients. The questionnaire could also serve as a bench-marking tool for palliative care, too.

Future research should validate the SNPC in more diverse populations (e.g., non-cancer patients, younger individuals, and different cultural or religious groups), conduct longitudinal studies to track changes in needs over the illness trajectory, and explore implementation strategies to integrate the SNPC into routine care while evaluating its impact on patient well-being, care satisfaction, and health system outcomes. We also think that further studies should be carried using qualitative methodology about spiritual and existential issues among younger patients in palliative care. This might widen the conceptualization and understanding the true spirituality from more narrow view using predefined items only.

## Conclusion

The SNPC is a valid and culturally sensitive instrument that captures the multifaceted spiritual needs of Finnish palliative care patients. By bridging the gap between patient-reported needs and clinical practice, it offers a practical tool for advancing holistic, patient-centered care. Future research should evaluate its longitudinal impact on outcomes and explore its integration into professional training and health-care systems.

## Supporting information

10.1017/S1478951526102168.sm001 Goyarrola et al. supplementary material 1 Goyarrola et al. supplementary material

10.1017/S1478951526102168.sm002 Goyarrola et al. supplementary material 2 Goyarrola et al. supplementary material

## Supplementary material

The supplementary material for this article can be found at https://doi.org/10.1017/S1478951526102168.
