# Supplementary material for: Development and validation of the questionnaire “Spiritual Needs in Palliative Care” in Finnish
Source: Palliat Support Care. 2026 Apr 7;24:e98. doi: 10.1017/S1478951526102168 (PMC13166331; doi:10.1017/S1478951526102168)
Supplement: Goyarrola et al. supplementary material 1 — Goyarrola et al. supplementary material [file S1478951526102168sup001.docx]

# Additional Information Form Research Number: ________

*Please complete the following questionnaire as accurately as possible*

*by writing or marking (X) the appropriate boxes.*

1. **Age**: _____ years

2. **Sex**: ___ Female ___ Male ___ Other

3. **What illnesses do you have?**

a) ___ Cancer – specify type: ____________________________

b) ___ Heart disease

c) ___ Pulmonary disease

d) ___ Neurological disease (e.g., ALS, multiple sclerosis)

e) ___ Other disease – specify: ____________________________

4**. Have you received a palliative care** plan (i.e., a treatment plan focused on symptom management)?

__ Yes __ No __ I don’t know

5. **How would you describe your general state of health during the past week?**

1……2……3……4……5……6……7 (1 = Very poor) (7 = Excellent)

6. **How would you rate your quality of life during the past week?**

1……2……3……4……5……6……7 (1 = Very poor) (7 = Excellent)

7. **Functional status (WHO)**: Circle the option that best describes your current level of functioning.

0. Able to carry out normal activities. Performs all pre-illness duties without restriction.

1. Restricted in physically strenuous activity but ambulatory and able to carry out light work.

2. Ambulatory and capable of all self-care but unable to carry out any work activities. Up and about more than 50% of waking hours.

3. Capable of only limited self-care; confined to bed or chair more than 50% of waking hours.

4. Completely disabled; cannot carry on any self-care; totally confined to bed or chair.

8. **Religious or worldview orientation**: Select the option that best describes you.

___ Christian, Lutheran

___ Christian, Orthodox

___ Christian, Catholic

___ Christian, other denomination: ________________________

___ Muslim

___ Buddhist

___ Other religion: ________________________

___ No religious or spiritual conviction

___ Spiritual but not affiliated with any religious community*

**In this study, spirituality is broadly defined as the pursuit of sacredness or meaning in life, whether religious or non-religious in nature.*
