# Supplementary material for: Development and validation of the questionnaire “Spiritual Needs in Palliative Care” in Finnish
Source: Palliat Support Care. 2026 Apr 7;24:e98. doi: 10.1017/S1478951526102168 (PMC13166331; doi:10.1017/S1478951526102168)
Supplement: Goyarrola et al. supplementary material 2 — Goyarrola et al. supplementary material [file S1478951526102168sup002.docx]

| **Hengellinen/henkinen tarve**  **Spiritual need** | **Miten tärkeä tarve on Sinulle?**  How important is this need to you? | | | | | **Miten tarve toteutuu kohdallasi tällä hetkellä?**  How is this need being met for you at the moment? | | | | |
| --- | --- | --- | --- | --- | --- | --- | --- | --- | --- | --- |
|  | En osaa sanoa  I cannot say | Ei lainkaan  tärkeä  Not at all important | Jossain määrin tärkeä  Somewhat important | Melko  tärkeä  Fairly  important | Erittäin  tärkeä  Very important | En osaa sanoa  I cannot say | Ei lainkaan  Not at all | Jossain määrin  To some extent | Melko hyvin  Fairly well | Erittäin hyvin  Very well |
| 1.Olla hyväksytty ja rakastettu sellaisena kuin olen  To be accepted and loved as I am |  |  |  |  |  |  |  |  |  |  |
| 2. Tulla kuulluksi ja ymmärretyksi  To be heard and understood |  |  |  |  |  |  |  |  |  |  |
| 3. Selviytyminen kärsimyksen kanssa  Coping with suffering |  |  |  |  |  |  |  |  |  |  |
| 4. Selviytyminen pelon ja huolien kanssa  Coping with fear and worries |  |  |  |  |  |  |  |  |  |  |
| 5. Mielenrauha  Peace of mind |  |  |  |  |  |  |  |  |  |  |
| 6. Vapaus häpeästä  Freedom from shame |  |  |  |  |  |  |  |  |  |  |
| 7. Vapaus syyllisyydestä  Freedom from guilt |  |  |  |  |  |  |  |  |  |  |
| 8. Ajatusten, tunteiden ja elämänkokemusten jakaminen läheisteni kanssa  Sharing thoughts, feelings, and life experiences with my closest ones |  |  |  |  |  |  |  |  |  |  |
| 9. Kokemus elämäni jatkumisesta läheisteni mielissä ja sydämissä  The experience that my life continues in the minds and hearts of my closest ones |  |  |  |  |  |  |  |  |  |  |
| 10. Kokemus elämän merkityksellisyydestä  The experience of life’s meaningfulness |  |  |  |  |  |  |  |  |  |  |
| 11. Kokemus, että kärsimyksellä on tarkoitus/merkitys  The experience that suffering has a purpose/meaning |  |  |  |  |  |  |  |  |  |  |
| 12. Toivo  Hope |  |  |  |  |  |  |  |  |  |  |
| 13. Saada anteeksi toisilta ihmisiltä  To receive forgiveness from other people |  |  |  |  |  |  |  |  |  |  |
| 14. Antaa anteeksi  To give forgiveness |  |  |  |  |  |  |  |  |  |  |
| 15. Osallistua hoitoani koskeviin päätöksiin  To participate in decisions concerning my care |  |  |  |  |  |  |  |  |  |  |
| 16. Puhua kuolemaan liittyvistä asioista  To talk about matters related to death |  |  |  |  |  |  |  |  |  |  |
| 17. Kokea luonnon kauneus tai yhteys luontoon  To experience the beauty of nature or a connection to nature |  |  |  |  |  |  |  |  |  |  |
| 18. Kokea turvallisuutta hoidossa  To feel safe in care |  |  |  |  |  |  |  |  |  |  |
| 19. Suhde Jumalaan tai johonkin korkeampaan voimaan  A relationship with God or a higher power |  |  |  |  |  |  |  |  |  |  |
| 20. Saada anteeksi Jumalalta tai joltakin korkeammalta voimalta  To receive forgiveness from God or a higher power |  |  |  |  |  |  |  |  |  |  |
| 21. Aikaa hiljaisuudelle, mietiskelylle ja/tai meditaatiolle  Time for silence, reflection, and/or meditation |  |  |  |  |  |  |  |  |  |  |
| 22. Aikaa rukoukselle  Time for prayer |  |  |  |  |  |  |  |  |  |  |
| 23. Papin tai muun uskonyhteisöni jäsenen tapaaminen  Meeting with a priest or another member of my faith community |  |  |  |  |  |  |  |  |  |  |
| 24. Osallistuminen uskonnollisiin toimituksiin  Participation in religious ceremonies |  |  |  |  |  |  |  |  |  |  |
| 25. Rukoilla yhdessä tai tietää, että puolestani rukoillaan  To pray together or to know that others pray for me |  |  |  |  |  |  |  |  |  |  |
| 26. Kokea läheisteni läsnäolo ja tuki kuoleman lähestyessä  To experience the presence and support of my closest ones as death approaches |  |  |  |  |  |  |  |  |  |  |
| 27. Osallistua omien hautajaisteni suunnitteluun  To participate in planning my own funeral |  |  |  |  |  |  |  |  |  |  |
| 28. Saada lohtua taiteista (esim. elokuvat, kirjat, musiikki)  To find comfort in the arts (e.g., films, books, music) |  |  |  |  |  |  |  |  |  |  |

Kuka täytti lomakkeen? Valitse sopiva vaihtoehto rastilla:

( ) täytin itse lomakkeen ( ) haastattelija täytti lomakkeen

Who filled in the form? Choose the appropriate option with a check mark:

( ) I filled in the form myself ( ) The interviewer filled it in

Mitä muita tarpeita Sinulla on? Kerro haastattelijalle tai kirjoita tähän.

What other needs do you have? Tell the interviewer or write them here

SNPC-FIN

Laita rasti (X) sopivaan kohtaan ruudukon sarakkeessa. Vastaa sekä tarpeen tärkeyteen että toteutumiseen!

Voit myös ympäröidä tarpeen, johon toivoisit erityisesti kiinnitettävän huomiota
